# Survey of the Impact of Decision Support in Preoperative Management of Anemia (i-Anemia): Survey Study

**DOI:** 10.2196/49186

**Published:** 2023-12-01

**Authors:** Gaëtan Mignanelli, Richard Boyer, Nicolas Bonifas, Emmanuel Rineau, Yassine Moussali, Morgan Le Guen

**Affiliations:** 1 Department of Anesthesia and Pain Medicine Hôpital Foch Université Versailles Saint Quentin Neuilly sur Seine France; 2 Department of Anesthesiology Weill Cornell Medical College New York, NY United States; 3 Ecole Polytechnique Palaiseau France; 4 Department of Anesthesia and Critical Care Angers University Hospital University of Angers Angers France; 5 Department of Anaesthesia Claude Galien Clinic Quincy-sous-Sénart France; 6 Department of Anesthesia and Pain Medicine Hôpital Foch Université Versailles Saint Quentin Suresnes France

**Keywords:** anemia, transfusion, patient blood management, preoperative optimization, preoperative, blood, decision support, randomized, case, survey, anesthesiologists, anesthesiologist, anesthesia, anesthesiology, professional development, digital health, surgery, perioperative

## Abstract

**Background:**

Major surgery on patients with anemia has demonstrated an increased risk of perioperative blood transfusions and postoperative morbidity and mortality. Recent studies have shown that integrating preoperative anemia treatment as a component of perioperative blood management may reduce blood product utilization and improve outcomes in both cardiac and noncardiac surgery. However, outpatient management of anemia falls outside of daily practice for most anesthesiologists and is probably weakly understood.

**Objective:**

We conducted a simulated case survey with anesthesiologists to accomplish the following aims: (1) evaluate the baseline knowledge of the preoperative optimization of anemia and (2) determine the impact of real-time clinical decision support on anemia management.

**Methods:**

We sent a digital survey (i-Anemia) to members of the French Society of Anaesthesia and Critical Care. The i-Anemia survey contained 7 simulated case vignettes, each describing a patient’s brief clinical history and containing up to 3 multiple-choice questions related to preoperative anemia management (12 questions in total). The cases concerned potential situations of preoperative anemia and were created and validated with a committee of patient blood management experts. Correct answers were determined by the current guidelines or by expert consensus. Eligible participants were randomly assigned to control or decision support groups. In the decision support group, the primary outcome measured was the correct response rate.

**Results:**

Overall, 1123 participants were enrolled and randomly divided into control (n=568) and decision support (n=555) groups. Among them, 763 participants fully responded to the survey. We obtained a complete response rate of 65.6% (n=364) in the group receiving cognitive aid and 70.2% (n=399) in the group without assistance. The mean duration of response was 10.2 (SD 6.8) minutes versus 7.8 (SD 5) minutes for the decision support and control groups, respectively (*P*<.001). The score significantly improved with cognitive aid (mean 10.3 out of 12, SD 2.1) in comparison to standard care (mean 6.2 out of 12, SD 2.1; *P*<.001).

**Conclusions:**

Management strategies to optimize preoperative anemia are not fully known and applied by anesthesiologists in daily practice despite their clinical importance. However, adding a decision support tool can significantly improve patient care by reminding practitioners of current recommendations.

## Introduction

Several studies have demonstrated the harmful aspects of blood transfusion during hospitalization. In the perioperative period and in a critical care setting, it was associated with risks and additional costs without certain benefit in the absence of active bleeding [1,2]. These analyses led to a restriction in red blood cell (RBC) prescriptions and a parallel effort to control bleeding and to determine restrictive hemoglobin thresholds for RBC transfusion. Current guidelines suggest a trigger of 7-8 g/dL, with studies consistently demonstrating noninferior or superior outcomes compared to more liberal approaches (eg, hemoglobin triggers of 9-10 g/dL) [3-7].

However, anemia has been identified as a modifiable risk factor for poor perioperative outcomes [8,9]. Preoperative anemia is relatively common, affecting 25% to 75% of patients with an increasing prevalence in older patients and those with cancer [10,11]. Consequently, the relationship between anemia and perioperative transfusion provides a related risk for perioperative morbidity and mortality [12-15]. Management of preoperative anemia has become a goal for the anesthesiologist before surgery, with some recommendations endorsing anemia treatment in patients undergoing surgery [16,17]. Patient blood management (PBM), as defined by the Society for the Advancement of Blood Management, is the timely application of evidence-based medical and surgical concepts designed to maintain hemoglobin concentrations, optimize hemostasis, and minimize blood loss in an effort to improve patient outcomes [18]. There are 3 categories of actions that describe PBM in practice: the optimization of RBC mass, the reduction of blood loss and bleeding, and the optimization of the patient’s physiological tolerance toward anemia [19]. Patient-centered decision-making is crucial when determining an individualized management plan and involves communication of the risks and benefits. Assessment of iron status, storage, and synthetic capacity should be performed to offer the best therapeutic strategy, which requires common biological tests measuring factors such as serum iron level, ferritin level, transferrin saturation [20,21]. A preanesthesia visit gives the opportunity to check for the presence or absence of anemia or iron deficiency because it allows enough time to correct or improve this functional defect. However, the current literature is still unclear regarding various situations that anesthesiologists must commonly manage. Therefore, implementation and access to consensus are practical aspects of PBM. Development of digital tools is crucial for detection and helping the physician with making a decision. In the present situation, we can imagine a digital “decision aid” that combines different guidelines and possesses an actualized decision tree to optimize PBM. We wish to measure this knowledge through a national and digital audit to further develop such a tool through a digital device.

The objectives of this work were 2-fold. First, we aimed to evaluate if the strategies of preoperative and postoperative anemia optimization by anesthesiologists fitted with the current recommendations. Second, we aimed to determine the usefulness of decision support to face these frequent and diverse situations involving anemia.

## Methods

### Ethical Considerations

This study received Institutional Review Board approval (CERAR IRB00010254-2020-131 - June 16, 2020). All eligible participants submitted a digital form of informed consent.

### Participants

Voluntary clinicians were recruited in 3 ways to participate to this web-based questionnaire study. First, 2000 flyers with a QR code were distributed during the 3 days of the national congress of the French Society of Anaesthesia and Intensive Care (SFAR) in September 2021. Usually, this congress includes about 5000 to 6000 participants, including physicians, residents, and anesthetic nurses. Second, in the 2 months following the congress, we sent an email via the SFAR network to all members with a brief explanation and the same link. In parallel, the SFAR and the Association of Young Anesthesiologists (AJAR) shared the link on their social networks (Facebook, Instagram, and Twitter [subsequently rebranded as X]). All clinicians recruited were invited to share the link to the questionnaire with other anesthesiologists.

### Protocol and Web Questionnaire

Once on the website, care providers selected whether or not to participate in this study. The first web page explained the aim of the study and reminded the reader of inclusion and exclusion criteria. Participants included only French-speaking anesthesiologists, including senior on-going physicians, fellows, and residents. Assistants, specialized nurse anesthesiologists, and medical students were not allowed to participate in the study.

If the participant was eligible, a digital form of informed consent was submitted. There was a brief explanation of the study, which specified that only anesthesiologists could participate and that the results would be anonymized. They agreed that their data would be anonymously used. We also provided the reference number of the Institutional Review Board approval so the participants could check it if they wanted. Then, access to the questionnaire was opened via a public web portal using an internet-based polling software called Typeform [22,23], which allowed participants to anonymously complete the questionnaire from different devices or navigators. Only 1 access per participant was available based on the participant’s IP address. Once consent was obtained, the participants approved their email to finally be randomized. Some questions about demographics were asked at this step.

The survey was created and validated by a committee of PBM experts. The final survey included 7 short clinical case vignettes containing up to 3 multiple-choice questions each (totaling 12 questions). These cases specifically involved potential preoperative and postoperative anemia situations. Some of them were built according to existing guidelines, while others led to an open discussion due to a lack of adapted recommendations. Moreover, no questions about intraoperative management of patients with anemia or control of bleeding were proposed to focus on the perioperative course (Table 1). Following receipt of informed consent, participants were randomly and equally divided into 2 groups: one with decision support and one without (the control group). The decision support group benefited from cognitive assistance during the survey; this was not available to the control group. The cognitive assistance consisted of the current recommendations about anemia management. This assistance was only visible if an error was made in the clinician’s responses. The clinician then had the choice to select a different answer or not.

**Table 1:** Summary of the questionnaire’s questions and objectives.

Case	Subject of the question	Specialty	Objective	References
1	Definition of preoperative hemoglobin thresholds	Orthopedics	Define the basic knowledge	[17]
1	Treatment of anemia (iron therapy and EPO^a^)	Orthopedics	Define the management of anemia	[17]
1	Indication to change an oral iron therapy with bad tolerance by an intravenous therapy	Orthopedics	Management of side effects	[17]
2	Woman without anemia; no indication to begin any treatment	Gastrointestinal surgery	Define the basic knowledge	[24]
3	Poorly tolerated anemia; indication to transfuse 1-2 blood units	Gynecological surgery	Define the management of anemia	[25]
3	Indication to start IV^b^ iron therapy to restore the iron status	Gynecological surgery	Define the management of anemia	[17]
4	Indication to postpone surgery	Gastrointestinal surgery	Define the management of anemia	[25]
5	Definition of preoperative hemoglobin thresholds for patients with terminal renal failure	Chronic kidney failure	Define the basic knowledge	[26]
5	Treatment of anemia (iron therapy first, then EPO if persisting anemia)	Chronic kidney failure	Define the management of anemia	[26]
5	Stop supplementations before polycythemia (thrombosis risk)	Chronic kidney failure	Management of side effects	[26]
6	EPO and iron therapy before surgery	Urgent cardiac surgery	Define the management of anemia	[27]
7	IV iron therapy for persisting iron deficiency after 34 weeks of amenorrhea	Obstetrics	Define the management of anemia	[28]

^a^EPO: erythropoietin.

^b^IV: intravenous.

### Data Collected

The following demographic characteristics of the population were collected: age, experience, specialties, and site of practice (public, private, or mixed). The time to complete the survey was recorded. Moreover, the quality of the responses was analyzed (false or correct). Cognitive assistance consisted of the current recommendations about anemia management. In fact, if a participant in the decision support group gave a wrong answer, a window appeared showing the current recommendations and their references (Table 1). The participants then had the choice to follow the recommendations and change their answer or to continue with their original answer. In the control group, participants were not able to go back and change answers to previous questions during the study. At the end of the survey, scores were made available to the participants. Individual participant data will not be shared.

### Statistical Analysis

The number of participants was calculated to allow for interpretation of the results using parametric tools. The main hypothesis was superiority in the rate of correct responses among physicians in the decision support group, with a planned difference of 25%. The estimated rate of correct response in the control group was 66%. With an α score of 5% and a power of 90%, at least 110 physicians were required. Moreover, to ensure a national representation of experience and geography, we determined the required number of completed forms to be 500 out of 9000 active physicians.

The data are expressed as the median (IQR) and mean (SD) according to their nonnormal or normal distribution. The quality of the questionnaire was determined based on the percentage of correct responses, and the comparison was mainly performed using chi-square and Wilcoxon-Mann-Whitney tests, as appropriate. The analysis was performed using SPSS 12.0 (IBM).

## Results

**Figure 1:**
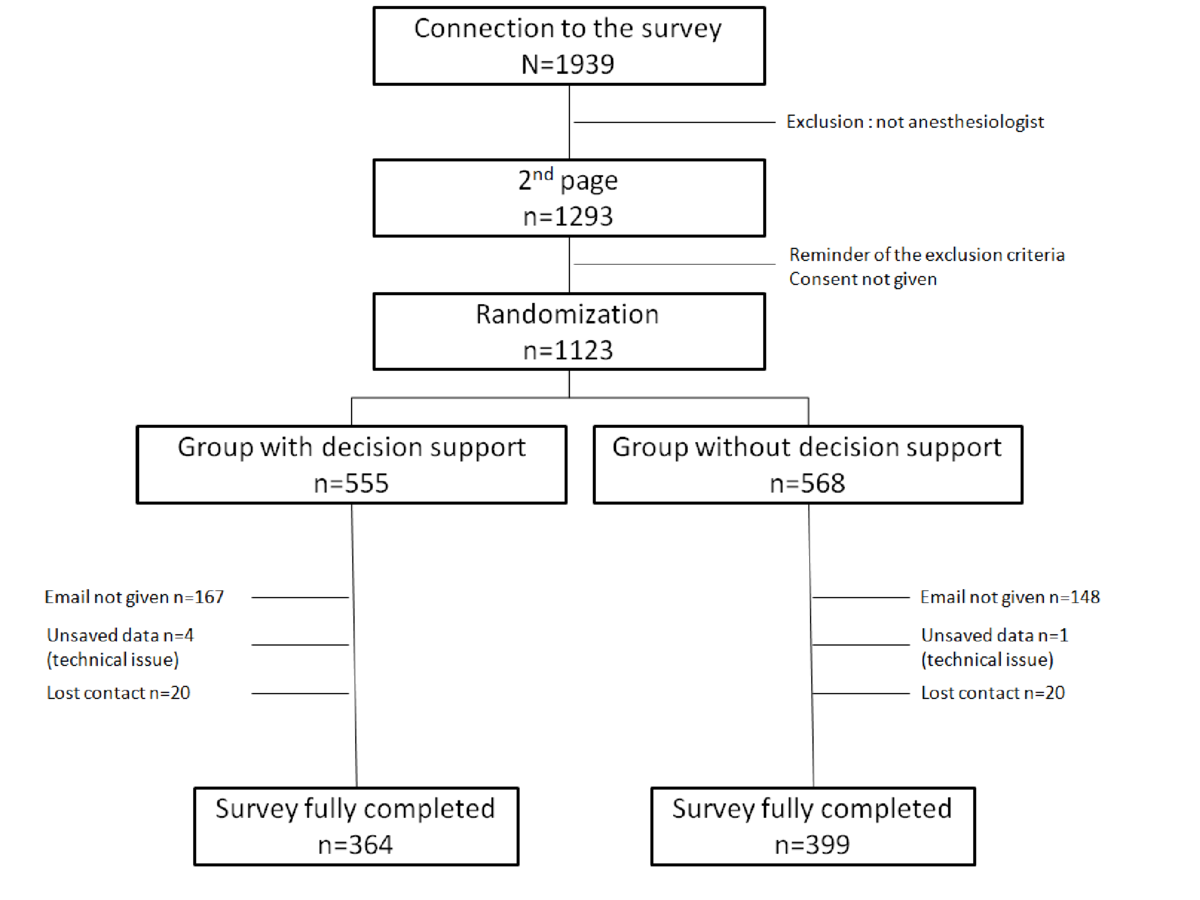
Flowchart of the i-Anemia participants.

In total, 1939 people opened the link, of which 1123 participants were randomized into the decision support and control groups before completing the questionnaire. Specifically, 555 (49.4%) anesthesia care providers were place in the decision support group receiving cognitive aid, and 568 (50.6%) were placed in the control group. The final cohort included 763 active French-native anesthesiologists representing the different fields of care (private and public). These participants included senior fellows and residents, as expected (Figure 1 and Table 2), for 763 questionnaires totally completed.

**Table 2:** Characteristics of the survey participants.

Characteristic, n (%)		All participants (n=1123)	With decision support (n=555)	Without decision support (n=568)
**Type of health care center**				
	University tertiary hospital	347 (30.9)	181 (16.1)	166 (14.8)
	Public secondary hospital	194 (17.3)	102 (9.1)	92 (8.2)
	Miscellaneous (cancer centers, mixed organization)	109 (9.7)	60 (5.3)	49 (4.4)
	Private care center	238 (21.2)	119 (10.6)	119 (10.6)
	Anesthesia residents	232 (20.7)	106 (9.5)	126 (11.2)
**Device used to participate**				
	Smartphone	804 (71.6)	380 (33.8)	424 (37.8)
	Computer	317 (28.2)	152 (13.5)	165 (14.7)
	Miscellaneous (eg, tablet)	2 (0.2)	2 (0.2)	0 (0)

The response rate was 65.6% (n=364) in the decision support group and 70.2% (n=399) in the control group *(P*=*.*49). Only respondents who fully completed the questionnaire were analyzed in the main outcome. The mean response times were 12.1 (SD 21.7) minutes and 9.2 (SD 23.5) minutes (*P*<.001) for the decision support and control groups, respectively. If we remove the outliers, defined as people who took more than 1 hour to complete the survey (n=5 in the decision support group and n=2 in the control group), the average time to answer was 10.2 (SD 6.8) minutes and 7.8 (SD 5.0) minutes (*P*<.001) for the decision support and control groups, respectively. The rate of correct responses was significantly higher in the decision support group (mean 10.3, SD 2.1) than in the control group (mean 6.2, SD 2.1; *P*<.001) (Table 3).

**Table 3:** Distribution of correct responses to each of the 12 multiple-choice questions for the decision support and control groups.

Question	Correct response rate, n (%)	
	Decision support (n=364)	Control (n=399)
Q1	321 (88.2)	221 (55.4)
Q2	295 (81)	178 (44.6)
Q3	309 (85)	241 (60.3)
Q4	320 (88.1)	306 (76.7)
Q5	350 (96.1)	363 (90.9)
Q6	350 (96.1)	288 (72.2)
Q7	298 (81.8)	146 (36.7)
Q8	331 (90.9)	133 (33.4)
Q9	307 (84.3)	20 (5.1)
Q10	312 (85.7)	235 (59)
Q11	318 (87.3)	128 (32.2)
Q12	319 (87.6)	150 (37.5)

Analyzing each multiple-choice question, the results were significantly better in the group with decision support (mean 87.7%, SD 4.6%) compared to the control group (mean 50.3%, SD 22.5%) (*P*<.001). Another interesting result was the relevant reduction in the variability of responses in the decision support group, which suggests that a guided response helps prevent participants from moving forward with a false response. This difference was more important in situations with unclear recommendations or in uncommon or specific cases (eg, questions 7-9 and 11-12). Question 7 was about postponing an elective surgery with mild risk of bleeding when anemia is discovered late (Multimedia Appendix 1). Questions 8 and 9 were about end stage renal disease (ESRD) and major surgery. Question 11 was about major vascular surgery, and question 12 asked about the use of intravenous iron therapy in obstetrics. These results show a great discrepancy between the two groups. The decision support group gave a more homogeneous response (*P*<.001), resulting in a better mean score compared to the control group (*P*<.001; Table 4). The magnitude of the difference was independent of the kind of institution and the experience level of anesthesiologist (senior or junior).

**Table 4:** Distribution of total scores out of 12 at the end of the survey for participants in the decision support and control groups.

Score	Participants, n (%)	
	Decision support (n=364)	Control (n=399)
0	2 (0.5)	0 (0)
1	1 (0.3)	3 (0.8)
2	0 (0)	11 (2.8)
3	0 (0)	24 (6)
4	2 (0.5)	43 (10.8)
5	7 (1.9)	66 (16.5)
6	4 (1.1)	68 (17)
7	19 (5.2)	80 (20.1)
8	19 (5.2)	55 (13.8)
9	31 (8.5)	32 (8)
10	57 (15.7)	11 (2.8)
11	80 (22)	6 (1.5)
12	142 (39)	1 (0.3)

## Discussion

### Principal Findings

This survey demonstrated that better performance in the management of preoperative anemia can be achieved if the anesthesiologists, regardless of experience, have at their disposal a cognitive aid (*P*<.001). Usually, guidelines require a certain time before being implemented, and decision support may accelerate this shift and help physicians make a decision, especially in rare situations or situations with a low level of prior knowledge.

The World Health Organization defines PBM as “a patient-focused, evidence-based, and systematic approach to optimize the management of patients and transfusion of blood products for quality and effective patient care. It is designed to improve patient outcomes through the safe and rational use of blood and blood products and by minimizing unnecessary exposure to blood products” [29]. Indeed, the incidence of preoperative anemia (about 30% regardless of the type of surgery) is critically important and increases with age. Additionally, preoperative anemia, which represents an independent risk factor of morbidity and mortality, may delay functional recuperation with a major risk of loss of autonomy in older patients [8,30]. The interest in the preoperative field is the potential reversibility of this factor if recognized with a different line of therapy, as included in different guidelines.

Despite their clinical and physiological fundamental importance, the strategies for optimizing the management of perioperative anemia are not always well-known and applied by anesthesiologists in daily practice. It is essential to understand the underlying reasons to improve practices and quality of care. This was the primary aim of the present study, and we observed weak knowledge of the recommendations among active anesthesiologists, with a mean rate of 6.2 (SD 2.1) out of 12 correct responses.

The second conclusion is the potential value of an immediately available cognitive aid, which significantly improved the rate of correct responses in our study. Several reasons may explain the moderate adherence to guidelines about perioperative anemia management. First, the importance of PBM is a relatively new topic that requires visibility and is not well-known by clinicians. In fact, the occurrence of complications in patients with anemia are mid-term or long-term complications, and the delay between general anesthesia and occurrence of complications may disconnect the physician from the patient’s daily analysis and prescriptions. Moreover, at the time of our study, some situations had been noted in the last recommendation, especially in the Frankfurt Consensus Conference published in 2018, and there was probably a need for updating to include additional situations, such as chronic renal failure and functional surgery [17,25]. Finally, strategies of PBM take time to be fully efficient, sometimes requiring a few weeks, and if the experts suggest postponing an elective surgery to correct iron deficiency or anemia, this is still difficult in many departments due to the typical course of patient care, the need to respect surgical planning, and the patient’s needs and wishes. To postpone an elective surgery remains difficult if the parties involved (eg, surgeon and anesthesiologist) do not share the same view of the importance of anemia management, and this is still the case in many teams. Furthermore, these strategies represent additional costs in the patients’ pathway, even if they reduce morbidity and mortality on balance [31].

The other aspect concerns the implementation of a cognitive aid. On one hand, there is communication work to be carried out to formulate clear and applicable recommendations in daily practice, which is a work in progress [32]. On the other hand, the addition of a decision support tool significantly improved patient management by reminding practitioners of the current recommendations. A simple line of work to help improve the quality of care could then be an implementation of these kinds of aids within the software used by anesthesiologists for their consultations, for example. Interest in such a solution was recently explored regarding the decision to perform transfusion. One single center randomized controlled trial compared young physicians who received computerized decision support about transfusion to those who did not [33] and showed an increase in the appropriate transfusion rate (RBCs, platelets, fresh frozen plasma) in the computerized decision support group (40.4%) compared with the control group (32.5%; risk ratio 1.24, 95% CI 1.13-1.37). Additionally, 3 cohort studies assessed RBC usage before and after the intervention [34-36] and showed a significant reduction in overall or inappropriate RBC usage (RBC transfusions per 100 inpatient days, *P*<.001) after computerized decision support was implemented. A statistically significant reduction in overall or inappropriate RBC usage over time (*P*=.01) was also reported. Furthermore, reduced 30-day re-admission (5.2%; risk ratio 0.62, 95% CI 0.56-0.69) and mortality (2.2%; risk ratio 0.60, 95% CI 0.51-0.71) were found in one single center trial. This cognitive reminder could be applied not only to anesthesiologists but to surgeons or any specialists involved in the medical management of the patients to share the crucial role of optimization. In view of its demonstrable benefits, there is an increasing awareness of the need to integrate the pillars of PBM within routine surgical care. In the United States, PBM has been successfully introduced in some centers [37], while in western Australia, it has become the standard of care [38]. PBM initiatives in Europe, however, have been variable and inconsistent, reflecting the difficulties that can be met with its implementation [39]. Multiple barriers, including lack of knowledge, interdisciplinary commitment, resources, and concerns, limit the translation of PBM guidelines into clinical practice [33-35,40]. Education and training should be considered to increase awareness of the clinical implications of anemia and the need for alternatives to transfusion. Typically, the lowest rate of correct responses in the control group concerned the management of anemia in patients with chronic kidney failure experiencing some trouble with iron supplements, erythropoietin, and situations with hemoglobin levels greater than 11 g/dL and high ferritin. Other cases with a low rate of correct responses were orthopedic cases and the management of anemia for pregnant women. Certain situations are well-described in the guidelines, but some patients fall in grey areas, warranting the use of cognitive aids to make the most pertinent choice. Once any deficiencies in the underlying knowledge have been addressed, attitude and behavior should change [41]. All recommendations and standard operating procedures must be easily accessible and aimed at supporting clinical judgment as the cornerstone of patient care.

The main population who completed our survey was composed of 763 active anesthesiologists; this is a large sample size with a high rate of complete answers and represents all our care areas and experience levels. This was permitted by using different channels of diffusion. We created the cases with the help of experts in PBM and made them representative of real-life, everyday clinical practice, which is an important strength.

However, there are several limitations of our study. The questionnaire study was destined only for French participants. Furthermore, there was a bias in participant recruitment, and we can assume a worse rate of correct responses among nonresponding physicians or incomplete questionnaires. Third, we did not track the rate of response changes in the decision support group to objectively measure the effect of receiving help. Additionally, we could not verify that all the participants truly were anesthesiologists; if they declared that they were we approved their participation without any further control. Finally, recommendations evolve quickly, which may have misled some participants.

### Conclusion

The recommendations about current PBM are not well-known by practicing physicians. We found that the implementation of decision support is very useful to face frequent and diverse situations of anemia and to improve the quality of patient management. Collaborative and continuous efforts to translate PBM guidelines into clinical practice, if done in an engaged, multidisciplinary, organized, and structured way, could make PBM the norm.

## Appendix

Multimedia Appendix 1 Case reports and questions.

## Abbreviations

PBM: patient blood management
RBC: red blood cell
SFAR: French Society of Anaesthesia and Intensive Care
