# Supplementary material for: Survey of the Impact of Decision Support in Preoperative Management of Anemia (i-Anemia): Survey Study
Source: JMIR Perioper Med. 2023 Dec 1;6:e49186. doi: 10.2196/49186 (PMC10724811; doi:10.2196/49186)
Supplement: Multimedia Appendix 1 [file periop_v6i1e49186_app1.docx]

Case reports and questions

**Case n°1** : Pre-anesthesia visit for a 63 year old man before a total knee prosthesis scheduled in 30 days. Comorbidities are arterial hypertension which is well controlled with an ACE inhibitor, disabling osteoarthritis predominant in the knees and obesity (BMI 32 kg.m^-2^). The physical exam presents no abnormality and no difficult airway management. Biology: Hemoglobin : 125g/L ; MCV 85fl ; Ferritin 40 ng/mL (N : 30-300ng/mL) ; Transferrin coefficient saturation 36% (N : 20-50%).

3 questions were about the pre-operative hemoglobin thresholds, definition of anemia and ways to control it (Q1-Q3).

**Case n°2 :** Pre-anesthesia visit for a 36 year old woman scheduled 3 days later for an elective cholecystectomy following a cholecystis, one month before. No comorbidities and a gynecologic history with a patient: G3P2 with vaginal delivery (a 8 years old boy and a 3 month girl). The last biological assessment shows : Hb 122g/L ; MCV 87fl ; CRP very low ; B-HCG negative.

One question about the pre-operative strategy of PBM (Q4).

**Case n°3** : Pre-anesthesia visit for a 58 years-old woman for an elective polymyomectomy justified by menometrorrhagia causing anemia. The surgery is planned in 2 weeks. On physical examination, the patient is asthenic, has cutaneous-mucous palor, heart rate at 110 bpm and respiratory rate at 20/min. The biological assessment prescribed by the gynecologist shows an anemia at 55/65 g/L with a 75fl MCV, 12% transferrin saturation coefficient and 7ng/mL ferritin. The rest of the laboratory tests has no particularity.

Two questions about the PBM (Q5-6).

**Case 4 :** Pre-anesthesia visit for a 63 year old man scheduled for abdominal surgery related to an ulcerative colitis with multiple episodes of flare-ups. The last event was one month ago and required a stay in the intensive care unit for abundant rectal bleeding with need of transfusion (8 RCB, 6 FFP). The patient now has one diarrhea per day without any rectal bleeding with medical treatment anti-TNFa and systemic corticosteroids. Furthermore, his medical history is constituted of high blood pressure controlled with ICE and thiazide-type diuretics, peripheral arterial occlusive disease with no physical limitation, myocardial infarction 3 years ago with stenting of the inter-ventricular artery, now treated with aspirin, Bêta-Blockers, ICE and statin. He doesn’t require any anticoagulation and had no symptoms in the last 3 years. The biological assessment yesterday showed : Hb 115g/L ; MCV 82fl ; Ferritin 80ng/mL ; No vitamin B9 and B12 deficiency ; CRP 12g/dL ; No kidney injury.

One questions about the PBM (Q7).

**Case n°5** : Pre-anesthesia visit for a 42 years-old patient with autosomal dominant polycystic kidney disease before elective embolization of an aneurysm the next month as part of a pre-transplant assessment. He has a kidney injury with glomerular filtration flow of 14mL/min, receives no treatment with EPO and no iron supplementation. He has no other background. The clinical examination doesn’t show any abnormality, with no asthenia. The last recent biological assessment showed : Hb 96g/L ; VGM 87fl ; Ferritinemy 80ng/mL.

Three questions about the PBM (Q8-10).

**Case n°6** : Pre-anesthesia visit for a 66 years old woman with an arterial malformation associated with thrombosis without peripheral perfusion disorder that must benefit from an aortic bypass. His medical history is : high blood pressure with triple therapy, non insulin-dependent diabetes. The biological assessment made the day before showed : Hb 114g/L ; MCV 90fl ; Ferritinemy 120ng/mL ; No vitamin B9, B12 and D deficiency ; No kidney injury ; CRP and PCT under threshold.

One question about the PBM (Q11).

**Case n°7** : Pre-anesthesia visit for a  29 years-old pregnant woman with her first child. She has no medical history and pregnancy behaving normally. Biological assessment of the 7th month : Hb 10,8g/L ; MCV 65fl ; Iron status showing a martial anemia. She is then treated with daily iron Per Os (Tardyferon 80mg) by the midwife. Biological assessment of the 8th month : Hb 10,8g/L ; MCV 63fl ; Ferritinemy 27ng/mL ; Transferrin coefficient saturation 25%. Supplementation with iron PO was well tolerated, and delivery was expected in 14 days.

One question about PBM (Q12).
